# A time-indexed reference standard of adverse drug reactions

**DOI:** 10.1038/sdata.2014.43

**Published:** 2014-11-11

**Authors:** Rave Harpaz, David Odgers, Greg Gaskin, William DuMouchel, Rainer Winnenburg, Olivier Bodenreider, Anna Ripple, Ana Szarfman, Alfred Sorbello, Eric Horvitz, Ryen W. White, Nigam H. Shah

**Affiliations:** 1 Center for Biomedical Informatics Research, Stanford University, Stanford, California 94305, USA; 2 Oracle Health Sciences, Bedford, Massachusetts 01730, USA; 3 National Library of Medicine, NIH, Bethesda, Maryland 20894, USA; 4 U.S. FDA, Silver Spring, Maryland 20993, USA; 5 Microsoft Research, Redmond, Washington 98052, USA

**Keywords:** Data mining, Health care, Outcomes research

## Abstract

Undetected adverse drug reactions (ADRs) pose a major burden on the health system. Data mining methodologies designed to identify signals of novel ADRs are of deep importance for drug safety surveillance. The development and evaluation of these methodologies requires proper reference benchmarks. While progress has recently been made in developing such benchmarks, our understanding of the performance characteristics of the data mining methodologies is limited because existing benchmarks do not support prospective performance evaluations. We address this shortcoming by providing a reference standard to support prospective performance evaluations. The reference standard was systematically curated from drug labeling revisions, such as new warnings, which were issued and communicated by the US Food and Drug Administration in 2013. The reference standard includes 62 positive test cases and 75 negative controls, and covers 44 drugs and 38 events. We provide usage guidance and empirical support for the reference standard by applying it to analyze two data sources commonly mined for drug safety surveillance.

## Background & Summary

The timely and accurate identification of adverse drug reactions (ADRs) during the post-approval phase is an important goal of the public health system. ADRs not captured during the clinical trials phase result in millions of injuries, hospitalizations, and deaths each year, and add billions of dollars to healthcare costs^1–4^.

Data mining approaches that enable drug safety researchers to analyze large volumes of data and to generate hypotheses (signals) of post-approval ADRs, play a key role in drug safety surveillance (pharmacovigilance)^4–6^. Strong signals may undergo expert adjudication, and such deliberation can lead to regulatory actions such as a drug withdrawal from the market or the issuance of public warnings.

Data mining for pharmacovigilance has predominantly relied on spontaneous reporting systems (SRS), such as the US Food and Drug Administration (FDA) Adverse Event Reporting System (FAERS)^4,5,7^, which pool reports of suspected ADRs collected from manufacturers, healthcare professionals, and consumers. Well-recognized limitations with existing surveillance approaches, including those based on SRS^4,6,8–10^, have motivated research into the systematic analysis (and mining) of additional data sources including electronic health records^8,9,11–13^, the biomedical literature^14–16^, social media (e.g., health forums and social networks)^17,18^, logs of information seeking activities on Web search engines^10,19^, and the use of chemical/biological knowledgebases^20,21^, in efforts to extend capabilities for pharmacovigilance and create a more holistic and robust system.

Regardless of the data source, a central challenge in drug safety research is the need for, and until recently the lack of, publically available, credible, and sufficiently large reference standards (‘ground truth’) to properly evaluate the performance characteristics of the data mining algorithms. Given that the complete set of true effects for all drugs is unknown, a definitive ‘gold standard’ of drug effects does not exist, and thus reference standards are used as surrogates.

A reference standard consists of a set of positive test cases, drug-event pairs recognized as truly associated (true ADRs), and a set of negative controls consisting of drug-event pairs that are highly unlikely to be associated. The inclusion criteria into each of the two sets is typically based on one or more supporting sources such as information on product (drug) labels, the scientific literature, spontaneous reports, authoritative online resources, or other medical resources. The reference standard should be large and diverse with multiple types of drugs and events to ensure generalizability.

To date, the limited availability of such benchmarks can be attributed to the domain expertise and substantial (typically manual) effort required for evidence gathering, identifying candidate test cases, ascertaining or refuting causality for each test case, and identifying the time-frame in which ADRs become publicly known.

Earlier efforts to develop reference standards were usually not systematic or transparent about their decision process, were limited in the size and diversity of drug-outcome pairs included, or lacked negative controls^5^. Recent efforts to develop reference standards, namely those of the OMOP^22^ and the EU-ADR^23^ projects, sparked substantial progress in the field, and have been applied to various data sources^5,11,12,22,24^. However, both reference sets were designed for the retrospective evaluation of ADR identification based on health records. It is argued that benchmarks based on ADRs that are well-known during the time-frame of evaluation (hence retrospective) do not reflect the desired ‘real-world’ performance characteristics of data mining methodologies—namely the ability to detect emerging or unknown ADRs^25^. Further, the public availability of knowledge about ADRs may affect, for example, spontaneous reporting rates for those ADRs or prescription patterns, which in turn could bias retrospective evaluations. Finally, the need for estimating the *time to detection* is not supported by existing reference standards.

We provide a first of its kind reference standard to support *prospective* performance evaluations, including lead time to detection, whose applicability is not restricted to the mining of a particular data source. The reference standard was systematically curated from all product label updates (e.g., warnings) communicated by the FDA in 2013. It includes 62 positive test cases and 75 negative controls, and covers 44 drugs and 38 events ranging from mild to rare and serious. We provide usage guidance for and demonstrate the utility of the reference standard in mining biomedical literature and FAERS.

## Methods

Product labels are an authoritative source of information about the risks, benefits, and pharmacological properties of drugs. A product label includes several sections that describe or list adverse events. Hundreds of safety-related label changes are approved by the FDA each year, and are communicated by the FDA to the public through monthly summaries posted at the FDA’s MedWatch website^26^. Each safety-related label change is categorized as one or more of the following: *Boxed Warning*, *Contraindication*, *Warning* and *Precautions*, *Adverse Reactions*, and *Patient Package Inserts/Medication Guide*, which correspond to a subset of the different sections used to organize drug information in the label. The safety-related label changes are based on multiple evidence sources including animal, observational, and pharmacokinetic studies, clinical trials, spontaneous reports, and case studies. However, the process by which the evidence is synthesized and a labeling decision is made by FDA panels is not documented.

### Positive controls

The FDA’s MedWatch website was used to obtain the complete list of all approved safety-related label changes (revisions) in the year 2013, including information about the drug involved in each safety issue, the safety issue, the date of the label change, and the section of the label that was updated.

The label changes for each month in the MedWatch website are organized in a table (called the ‘summary view’) that lists the drug involved in a label change and the corresponding label section that was updated. Based on the summary view we considered only the following label changes: (1) those that were marked as updating at least one of the sections: Boxed Warning, Warning (or Precautions), and Adverse Reactions. (2) Those including orally administered drugs in the form of tablets or capsules. These two criteria represented the large majority of label changes. Strictly injectable drugs, creams, and sublingual drugs were not considered because of the difficulty associated with identifying appropriate negative controls of the same form.

**Figure 1:**
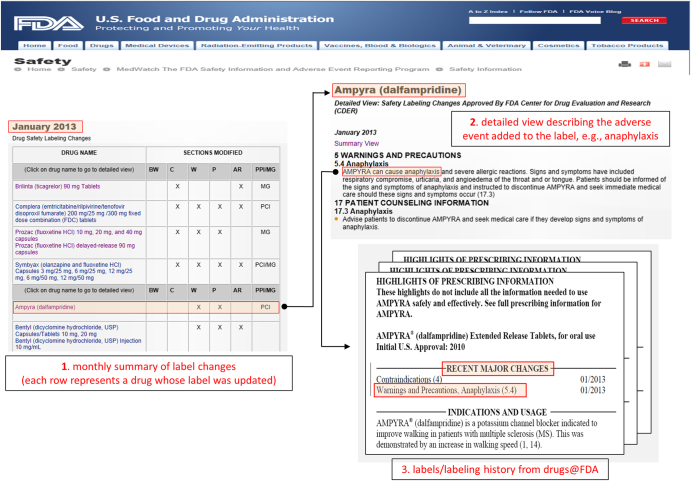
Illustration of the resources (from FDA’s MedWatch and Drugs@FDA) and the process used to extract, filter, and verify, candidate test cases for the reference standard. (1) Initial candidate test cases are obtained from FDA’s MedWatch monthly summaries, which provides a table of drugs (e.g., Ampyra) whose labels have been revised to include new safety information. (2) The events associated with each drug whose label was revised (e.g., Anaphylaxis) are obtained from the ‘detailed view’ linked to each row of a monthly summary Table. (3) The labels and revision history of each drug is obtained from Drugs@FDA to verify the labeling revision, and ensure that the candidate test case is indeed new and not qualified by special conditions.

The safety issue (event) associated with each drug (not specified in the summary view) was extracted from the safety summary narrative linked to each entry in the table (called the ‘detailed view’). The narrative usually includes several events, all of which were extracted. This extraction process was used to generate a set of quadruples 〈*drug*, *event*, *date*, *section*〉 that served as our initial set of candidate positive test cases to be included in the reference standard. Figure 1 illustrates the process and resources used in this first step.

The MedWatch website only provides a succinct summary of the label changes, not the actual labels that are necessary for further appraisal of each candidate test case. The labels for each drug, including their previous versions (from drug approval) were obtained from Drugs@FDA^27^—a FDA public database for US approved prescribed and over-the-counter drugs that includes the most recent drug label versions, labeling revisions, approval history, approval letters, reviews, and other information (Figure 1).

Each of the label changes (quadruples) was manually verified by inspecting its corresponding label, ensuring that the safety issue was clearly mentioned in the label (in the appropriate section), and that the label change dates matched those in Drugs@FDA.

Given that not all label changes are truly new, relevant, or appropriate for benchmarking data mining methods, the initial list of label changes (quadruples) was refined by applying 10 filtering rules. The rules, accompanied by the rationale for their use and examples are displayed in Table 1. Collectively, the rules are used to ensure that the positive test cases included in the reference standard are indeed new (to the year 2013), are difficult to dispute based on labeling information, and that each of the test cases (both negative and positive) can be appropriately analyzed/tested using common data mining methodologies across various data sources.

Of the 975 initial label changes (quadruples) the filtering resulted in a much smaller final set of 91 quadruples. This low number illustrates the difficulty and sparseness of data available for deriving reference standards of recently published ADRs that are necessary for prospective performance evaluations.

### Drug and event normalization

Drugs posted on the MedWatch website are specified either by their brand names (e.g., Prozac), or by their generic name (e.g., fluoxetine), and are often accompanied by strength (e.g., 10 mg), and dose form (e.g., tablet/capsule). Given that it is a drug’s active ingredient that provides its distinctive clinical properties (or potential to chemically induce an adverse reaction), we normalized (mapped) each drug in our final set of filtered quadruples to its active base ingredient via RxNorm^28^—a controlled vocabulary for generic and branded drugs that associates drugs with their ingredients, strength, and forms.

Drugs with more than one active ingredient (a total of five cases) were mapped to each of their active ingredients. In some cases (three of the five), the ingredient likely to be associated with the event could be inferred by identifying the ingredient common to a batch of label changes referring to the same adverse event. For example, the ingredient *olmesartan* could be inferred for the event ‘sprue-like enteropathy’ posted on the MedWatch website on July 2013 in relation to the drug *Benicar* (active ingredient: olmesartan) and in relation to the drug *Tribenzor* (active ingredients: olmesartan, amlodipine, hydrochlorothiazide).

Closely related events that are synonymous, that describe the same clinical syndrome, or have a common pathogenesis, were grouped and given a unique representative name, which we refer to as the ‘event concept name’. For example, the related events *choreoathetosis*, *dyskinesia*, *dystonia*, *movement disorders*, *oculogyric crisis* (all movement disorders) were grouped and assigned the event concept name ‘movement disorders’. The motivation for event normalization is three-fold: (1) Eliminate unnecessary redundancy due to synonymy or the heterogeneity of terms that may describe the same event; (2) simplify and enhance the identification and retrieval of cases (data records) needed for analysis, and; (3) address reporting artifacts on the MedWatch website (or label) whereby events that lead to one another and relate to the same clinical syndrome are often reported together. For example, *QT prolongation* may lead to *torsades de pointes* (a type of arrhythmia), both of which are typically reported together and thus assigned the event concept name ‘QT prolongation’. Another example includes the related events *peripheral vasculopathy* and *Raynaud’s phenomenon* (blood circulation disorders), which are often reported together and thus assigned the event concept name ‘peripheral vascular disorders’.

### Negative controls

The set of negative controls was created by randomly pairing a normalized drug that appears in the set of positive controls with a normalized event that appears in the set of positive controls, and manually verifying that each of the random pairs is not reported in the underlying drug’s label as an ADR. That is, based on labeling information the drug and event are not associated. This process was repeated until each drug or event appearing in the set of positive controls was matched with at least one negative control. In addition, we ensured that none of the drugs or events in the set of negative controls is over-represented with respect to the positive controls. Specifically, in order to maintain a balance between the distribution of drugs and events appearing in the two sets of test cases, we require that the difference between the number of occurrences of a specific drug or event in the negative controls and its number of occurrences in the positive controls be at most equal to one. Using the same set of drugs and events in each of the two sets of test cases, and maintaining a balanced distribution thereof, reduces the likelihood of a biased performance evaluation.

### Event definitions

To examine whether a drug and event are associated in an underlying data source, one needs to first identify the relevant cases (e.g., patient records, spontaneous reports, scientific articles) that refer to the drug or event in question. A medical concept (adverse event in this case) may manifest itself in data through different medical terms (or codes). Therefore, event (or outcome) definitions are used to aid in the identification and retrieval of potentially relevant cases from the data to support analysis. An *event definition* consists of a group of terms from a designated medical controlled vocabulary that relate to and are consistent with a description of the overall clinical syndrome associated with a particular adverse event. The included terms usually describe signs, symptoms, diseases, syndromes, physical findings, and laboratory test results.

The terms we used for each event definition were drawn from the Medical Dictionary for Regulatory Activities (MedDRA)^29^—a hierarchically organized terminology designed for ADR applications. The terms were restricted to the ‘Preferred Term’ level in the MedDRA hierarchy (the level used in most ADR applications). The scope of each of the event definition consists of two levels; *narrow*—highly specific terms more likely to represent the condition of interest, and *broad*—the narrow terms in addition to more general terms, which may assist in identifying more relevant cases at the cost of decreased precision. We recommend using the narrow level of definitions as the default option, but note that the broad level of definitions may be more appropriate for certain data sources.

The definitions were created and reviewed by several authors of this work with professional medical training. Though MedDRA is the default event terminology for our reference standard, the user can map our event definitions to an alternative terminology (e.g., the one used to encode the particular data source being mined) via publicly available resources such as the Unified Medical Language System (UMLS)^30^.

## Data Records

The reference standard is publicly available online at figshare (Data Citation 1) as an Excel spreadsheet including four worksheet tabs. The four worksheet tabs provide in tabular form: (1) the actual reference standard—drug-event pairs labeled with ground truth (marking the positive and negative test cases); (2) event definitions for case/record identification; (3) the drug ingredients used in the reference standard and their associated RxNorm codes, and; (4) labeling information for the positive test cases, such as the date of the labeling change and the section that was updated. This file provides data to enable the analysis of lead time to detection and analysis by labeling section.

The fields contained in each of the four worksheet tabs are as follows:

Tab 1—Reference Standard (3 columns, 137 rows, 62 positive+75 negative tests cases)

EVENT_CONCEPT_NAME: Name of the normalized event that comprises a test case (drug-event pair).

DRUG_CONCEPT_NAME: Name of the drug (active ingredient) that comprises a test case (drug-event pair).

TEST_CASE_STATUS: Label with the values of either 1 or 0 that indicates whether the test case is a positive control (labeled 1) or a negative control (labeled 0)

Tab 2—Event Definitions (5 columns, 545 rows)

EVENT_CONCEPT_NAME: Name of the normalized event that comprises a test case (drug-event pair).

MEDDRA_PT: A MedDRA preferred term that is part of the set of terms that define the event specified in the EVENT_CONCEPT_NAME field.

DEFINITION_LEVEL: Label with the values of either ‘narrow’ or ‘broad’ that determines whether the MedDRA term specified in the MEDDRA_PT field is part of the narrow or broad event definition.

MDR_CODE: MedDRA code associated with the term specified in the MEDDRA_PT field. The code is an alternative to the MedDRA term for case identification, and also used for terminology mapping purposes, e.g., mapping a MedDRA term to its equivalent MeSH heading when the data being mined is the biomedical literature (see usage notes). The MedDRA code also eliminates ambiguity with respect to MedDRA term specification.

UMLS_CUI: Unified Medical Language System (UMLS) Unique Concept Identifier (CUI) associated with the MedDRA term specified in the MEDDRA_PT field. As above, codes are used for case identification and for terminology mapping purposes.

Tab 3—Drugs (2 columns, 44 rows)

DRUG_CONCEPT_NAME: Name of the drug (active ingredient) included in the reference standard.

RXNORM_CODE: RxNorm code associated with the drug specified in the DRUG_CONCEPT_NAME field. The RxNorm code can be used instead of the drug name for case identification. The code can also be used for terminology mapping purposes, and eliminates ambiguity with respect to drug specification.

Tab 4—Labeling Information (8 columns, 62 rows)

EVENT_CONCEPT_NAME: Name of the normalized event that comprises a test case (drug-event pair).

DRUG_CONCEPT_NAME: Name of the drug (active ingredient) that comprises a test case (drug-event pair).

LABEL_CHANGE_MONTH: Month (in the year 2013) as a numeric value (1–12) for which an association (positive test case) became known according to product labels. When multiple labels referring to the same ingredient and event have been updated the earlier of the label change dates was selected.

YEAR_IMPLICATED_BRAND_APPROVED: Year in which the brand drug containing the implicated ingredient was approved. If multiple brand drugs contain the same ingredient, multiple approval years (separated by ‘/’) each corresponding to a different brand drug, are specified.

BW: Label with the values of either 1 or 0 indicating whether the association was mentioned in the ‘Boxed Warning’ section of the label.

W: Label with the values of either 1 or 0 indicating whether the association was mentioned in the ‘Warnings’ section of the label.

AR: Label with the values of either 1 or 0 indicating whether the association was mentioned in the ‘Adverse Reactions’ section of the label.

AR_POSTMARKETING: Label with the values of either 1 or 0 indicating whether the association was mentioned in the ‘Adverse Reactions/Postmarketing’ subsection of the label.

## Technical Validation

The use of labeling information about adverse events appears to be a valuable approach for deriving reference standards to evaluate pharmacovigilance data mining methodologies, and has formed the basis for several existing and commonly-applied reference standards such as the one created by the OMOP^22^. Each label change undergoes thorough review by FDA experts prior to its approval. Notwithstanding, it is stated that, for an adverse event to be included in a label, ‘there should be reasonable evidence of a causal association between the drug and the event, but a causal relationship need not have been definitively established’^31^. Establishing definitive causality for a given test case is beyond the scope of this work. Strengthening the support for each association in the reference standard can be achieved by gathering evidence from additional sources, e.g., the scientific literature, spontaneous reports, and health records. However, strengthening the support for each association by using additional sources would limit the utility of reference standard by excluding its use for assessing the included data source’s potential for ADR detection. Information from a specific data source (e.g., the biomedical literature) cannot directly be used to form a reference standard that will later be applied to evaluate data mining approaches on the same data source. Thus, the validation of our reference standard is limited to the process of its creation, rather than the validity of each test case. The validation of the process was performed using an iterative consensus-oriented approach. At least two authors of this manuscript independently performed each of the steps described in the methods to retain only those candidate test cases for which there was an agreement. The results of each step were then reviewed by several of the remaining authors and revised until the consent of all participants was obtained.

The next section (Usage Notes) provides additional empirical support for the validity as well as utility of the reference standard.

## Usage Notes

The large majority of data mining approaches for pharmacovigilance are designed to compute surrogate measures of statistical association between specific pairs of drugs and clinical outcomes from an underlying data source^4^. The data mining approaches differ with respect to the exact measures that are used and the statistical adjustments that are applied to account for small sample sizes and confounding factors. The association measures are typically used as signal scores, whereby larger signal scores represent stronger associations that are assumed more likely to represent true ADRs. Rankings of these signal scores or thresholds are then used to flag associations for further appraisal.

We applied our reference standard (via the supplied event definitions) to longitudinally assess the prospective performance of FAERS and MEDLINE as ADR signal detection sources. The area under the receiver operating characteristic curve (AUC) was used as our main performance index. Performance and other statistics were recorded at different pre-specified years (e.g., each year in the past five years) by using all time-stamped records available for a given data source up to the end of the pre-specified year.

By applying our reference standard to the mining of two commonly used data sources in pharmacovigilance, we provide guidance for its use in assessing other data sources or assessing novel signal detection methodologies. Through the results we demonstrate the utility of the reference standard and also provide empirical support toward its validity.

### FAERS

To date FAERS contains over 6 million spontaneous reports of suspected ADRs collected from healthcare professionals, consumers, and pharmaceutical companies. Each report includes one or more adverse events that appear to be associated with the administration of a drug, as well as indications and limited demographic information.

Because drug names reported in FAERS were available to us based on the Anatomical Therapeutic Chemical Classification System (ATC)^32^ coding system, we mapped the drugs in our reference standard, coded in RxNorm, to their ATC equivalents. Events in FAERS are specified using MedDRA Preferred Terms—the same terminology used for the event definitions of our reference standard. Thus, a FAERS report was considered to mention an event in our reference standard if any of the MedDRA terms defining that specific event were included in the FAERS report, in which case the report was then tagged with the corresponding event concept name. This same data transformation process was applied in a related study^5^ designed to evaluate the retrospective signal detection performance of FAERS based on the OMOP reference standard.

Signal scores for each test case in our reference standard were computed using the FDA’s primary signal detection algorithm called the Multi-item Gamma Poisson Shrinker (MGPS)^33^. The core metric calculated by MGPS is a ratio of the *observed* reporting rate of a drug-event pair to its *expected* rate under the assumption that the drug and event occur independently. To account for the uncertainty associated with small observed and expected counts, MGPS uses a Bayesian model to shrink the observed-to-expected ratio towards the baseline case of no association (value of 1). The MGPS signal score used in our evaluation is denoted by EBGM (empirical Bayes geometric mean).

Table 2 provides several statistics related to the prospective ADR detection accuracy of FAERS assessed at different time points (each of the years 2007–2013) using our reference standard. The narrow event definitions were used for this evaluation. For each time period, the table provides the signal detection accuracy (AUC), the median number of reports for the set of positive (N+) versus the set of negative (N−) test cases, and the median signal score for the set of positive (EBGM+) versus the set of negative (EBGM−) test cases. Based on all FAERS data available at the time of our analysis, that is, data up to 2013Q3 (potentially including reports received at the FDA after an association is communicated to the public via MedWatch), the application of data mining to FAERS (using MGPS) resulted in an AUC of 0.81. As expected, signal detection accuracy deteriorates as the lead time to detection increases, i.e., as we go further back in time in an attempt to detect signals earlier. The accuracy ranges from an AUC of 0.77 for a lead time of approximately one year from the date a label was changed (end of 2011), to a relatively modest AUC of 0.7 for a lead time of approximately five years (the end of 2007). The table also shows that the median number of reports submitted to the FDA, and the median signal score for the set of positive test cases is always larger than that of the set of negative test cases; which provides additional support for the validity of our reference standard.

### Biomedical literature

MEDLINE is the U.S. National Library of Medicine (NLM) publicly available electronic database of over 21 million citations to biomedicine and life sciences articles^34^. NLM indexers describe the content of articles by assigning key Medical Subject Headings (MeSH) that appear on the bibliographic citations in MEDLINE. The MeSH headings include main subject headings (with or without subheadings) and supplementary concepts and can be referred to as MeSH annotations. MEDLINE bibliographic citations can be downloaded from PubMed searches; the MeSH annotations can be extracted from the XML files of the bibliographic citations, stored in databases, and used to support various biomedical applications.

In the context of drug safety the subject (main) headings typically describe clinical conditions, drugs, or drug classes, whereas the subheadings narrow the scope to specific aspects of the main headings. For example, the main/subheading combinations ‘Cyclooxygenase Inhibitors/adverse effects’ and ‘Myocardial Infarction/chemically induced’, suggest that an article content is about the ADR relationship between COX-2 inhibitors and myocardial infarction.

We used the following MeSH query via PubMed to obtain approximately 360 K MEDLINE citations referencing drugs, adverse events, and a possible relationship between the two:

‘Chemicals and Drugs Category/adverse effects’[MeSH] AND (‘chemically induced’[Subheading] OR [main heading with ‘chemically induced’ connotation]),

where the latter is one out of the 19 pre-coordinated main headings we identified that do not require a ‘chemically induced’ subheading, such as ‘Drug-Induced Liver Injury’[MeSH].

For the sake of brevity, we omit details related to the extraction and processing of the MeSH annotations resulting from MEDLINE citations retrieved by this PubMed query, and refer the readers to similar work discussed by Avillach *et al.*^15^, and Shetty *et al.*^14^ To apply our reference standard we mapped the drugs of the reference standard (coded in RxNorm) to their MeSH equivalents. The event definitions of the reference standard, specified via MedDRA preferred terms, were also mapped to their MeSH equivalents. Similar to the processing of FAERS, MEDLINE articles whose MeSH annotations were part of an event definition were tagged with the corresponding ‘event concept name’.

Signal scores for each test case in our reference standard were computed based on the Odds Ratio (OR) measure of association with a zero-cell correction (adding 0.5 to each count in a 2×2 table that contains zero in any of its cells). Unlike the case of FAERS, there are no established methodologies for the mining of MEDLINE. However, more elaborate methods to compute signal scores, which take advantage of other information supplied by MeSH annotations (e.g., publication types, study types), are currently being investigated.

Table 3 provides statistics related the accuracy of ADR signal detection and the ADR information retrieval capacity of MEDLINE. The total number of articles processed is approximately 360,000. Following the approach discussed by Avillach *et al.*^15^, we also used the number of articles (N) whose MeSH annotations include a drug-event pair of interest (e.g., a test case in the reference standard) as a signal score. In this experiment (versus the one for FAERS) we also examined differences between the narrow and broad event definitions of our reference standard. Our results suggest that the use of number of articles as a predictor (signal score) provides greater accuracy than using the odds ratio as a signal score (AUC_N versus AUC_OR). The results also suggest that the use of the broad events definitions (versus narrow) provide increased accuracy for the mining of MEDLINE. Avillach *et al.*^15^ made use of three articles as a signal score threshold. We found that the use of one article as a threshold provides a substantial increase in accuracy as measured by the F-score (F@1 versus F@3). This finding underlies the need for reference standards such as ours, since it demonstrates that retrospective evaluations based on well recognized associations (as done by Avillach *et al.*), versus prospective evaluations based on relatively new ADRs can lead to different recommended methodologies. Lastly, the table shows that MEDLINE contains information (articles mentioning a specific drug-event pair) for a substantially larger number of positive test cases (M+) than negative test cases (M−). Given that MEDLINE is often used to assess or confirm causal relationships between drugs and medical outcomes, this finding provides increased support for the validity of the reference standard.

### Additional notes

Keeping such proactive reference standards up-to-date with new labeling revisions and current safety information requires significant, ongoing effort, and needs to be a community program such as that of the Observational Health Data Science Initiative (www.ohdsi.org), which recently undertook the task of creating a public knowledge base of all time-stamped ADRs^35^. In this respect, although we are putting forth a time-indexed reference standard and a new methodology for creating such reference standards, we realize that the relevance and value of such reference sets are time-sensitive in themselves, and future efforts should include the dates and periods of time for which the data was collected. It is also worth mentioning that the European Medicines Agency (EMA) maintains a similar resource^36^ to that of FDA’s MedWatch that can used to identify candidate test cases based on the Summary of Product Characteristics (SPC) of medicinal products authorized in the EU. Last, a word of caution when interpreting the dates attached to the positive test cases of our reference standard. These reflect the dates by which a label was revised and potentially the date when an association became known to the general public, but not the date by which initial internal investigations by FDA were undertaken. These can precede the labeling revision by up to two years.

## Additional information

**How to cite this article:** Harpaz, R. *et al.* A time-indexed reference standard of adverse drug reactions. *Sci. Data* 1:140043 doi: 10.1038/sdata.2014.43 (2014).

## Supplementary Material



## Figures and Tables

**Table 1:** Filtering rules used to select candidate positive test cases based on labeling revisions.

Filtering Rule, Rationale, and Examples
*Event listed in earlier label* *Rationale*: The association is not new *Example*: The event myalgia associated with the drug Arimidex posted at MedWatch on May 2013 as ‘added.....myalgia’ in the Adverse Reaction section, was mentioned in a labeling revision dated April 2009. Similarly, the event anaphylaxis associated with the drug Azulfidine EN posted on July 2013 under the Adverse Reactions section, was mentioned in a labeling revision dated December 2012
*Event part of a contraindication* *Rationale*: Associations where the event is qualified by a co-existing contraindicated situation (e.g., presence of a comorbid condition, physiological state, demographic risk factor, coadministered drug) require non-standard analyses approaches to properly identify or test because the co-existing contraindicated situation will also need to be identified and incorporated into the analysis *Example*: The association Depakene (Valproate)-hepatotoxicity posted on July 2013 as an update to the Warnings and Precautions section: ‘Valproate is contraindicated in patients known to have mitochondrial disorders caused by POLG mutations… Valproate-induced acute liver failure and liver-related deaths have been reported in patients with hereditary neurometabolic syndromes caused by mutations in the gene for mitochondrial DNA polymerase γ (POLG)…’
*Event is too general* *Rationale*: Challenging to accurately identify the cases (data records) needed for analysis *Example*: The event hypersensitivity posted on February 2013 in association with Aromasin (exemestane) is a broad term that refers to wide range of adverse reactions such Anaphylaxis, Thrombocytopenia, Lupus Nephritis, Montoux and more
*Events like drug abuse, misuse, dependence, and withdrawal* *Rationale*: Challenging to accurately identify the cases (data records) needed for analysis, e.g., drug abuse or withdrawal are not necessarily coded (billed for or mentioned) in health records and may need to be inferred via other mechanisms that are prone to error *Example*: Withdrawal effects associated with the drug Ambien (Zolpidem) posted on April 2013
*Multi-ingredient drugs where one of the ingredients is already known to cause the event* *Rationale*: The association is unlikely new given that the ingredient already known to be associated with the event is most likely the culprit *Example*: The association between Symbyax (olanzapine+fluoxetine) and the event QT Prolongation posted on July 2013. Fluoxetine (Prozac) is already known to be associated with the event QT Prolongation
*The event is inferred from other drugs with the same/similar ingredients to the drug in question* *Rationale*: The association is not new, and possibly not observed or reported with the specific drug in question *Example*: The association between Xyzal (levocetirizine) and myoclonus posted on November 2013; ‘Besides these events reported under treatment with XYZAL, other potentially severe adverse events have been reported from the post-marketing experience with cetirizine. Since levocetirizine is the principal pharmacologically active component of cetirizine, one should take into account the fact that the following adverse events could also potentially occur under treatment with XYZAL: orofacial dyskinesia, severe hypotension, cholestasis, glomerulonephritis, still birth, tic, myoclonus, and extrapyramidal symptoms’
*Events that seem to have been identified through or supported by pre-marketing clinical trials* *Rationale*: The association was observed in pre-marketing clinical trials and therefore unlikely newly observed *Example*: The association between Potiga (ezogabine) and retinal abnormalities posted September 2013; ‘The retinal abnormalities observed with POTIGA have been reported in patients who were originally enrolled in clinical trials with POTIGA’
*A different preparation or time release mechanism (IR versus ER) of the drug already known to be associated with the event* *Rationale*: Cannot definitively determine that the association is new *Example*: The association between Kapvay (Clonidine) extended-release and hallucinations posted on February 2013. A labeling revision from September 2010 states the following: ‘The following less frequent adverse reactions have also been reported in patients receiving immediate-release clonidine, but in many cases patients were receiving concomitant medication and a causal relationship has not been established… hallucinations’
*Associations included in existing reference standards* *Rationale*: The association is most likely not new. There is also a need to separate associations used in retrospective benchmarks from those designed for prospective benchmarks since the two types of benchmarks should complement each other *Example*: The association between Clozaril (clozapine) and liver injury posted on March 2013 is part of the OMOP reference standard (positive test case)
*The event is polymorphic to another event included in an earlier label* *Rationale*: The association is unlikely new *Example:* Torsades de pointes posted on July 2013 in association with Agrylin (anagrelide) is a type of tachycardia included in earlier label dated November 2011

**Table 2:** Statistics related to the prospective signal detection performance of FAERS.

		**Medians**			
**Year**	**AUC**	**N+**	**N−**	**EBGM+**	**EBGM−**
2013Q3	0.81	17	12	1.65	0.59
2012	0.82	17	11	1.65	0.55
2011	0.77	19	10	1.68	0.54
2010	0.75	17	9	1.66	0.55
2009	0.72	14	11	1.40	0.60
2008	0.70	13	10	1.56	0.62
2007	0.70	11	9	1.32	0.56
AUC, area under the receiver operating characteristic curve; EBGM+, median signal score for the set of positive test cases; EBGM−, median signal score for the set of negative test cases; N+, median number of reports for the set of positive test cases; N−, median number of reports for the set of positive negative cases.					

**Table 3:** Statistics related to the prospective signal detection performance of MEDLINE.

	**AUC_OR**	**AUC_N**	**F@3**	**F@1**	**M+**	**M−**
narrow 2013	0.67	0.73	0.34	0.65	33	6
narrow 2012	0.66	0.71	0.34	0.63	31	6
narrow 2011	0.66	0.71	0.34	0.63	31	6
narrow 2010	0.66	0.70	0.34	0.61	30	6
narrow 2009	0.63	0.68	0.29	0.57	27	5
broad 2013	0.71	0.75	0.47	0.70	40	13
broad 2012	0.71	0.74	0.47	0.68	38	12
broad 2011	0.70	0.73	0.45	0.67	37	12
broad 2010	0.70	0.74	0.44	0.67	37	11
broad 2009	0.67	0.71	0.40	0.63	33	10
AUC_N, area under the receiver operating characteristic curve for number of articles used as a signal score; AUC_OR, area under the receiver operating characteristic curve for the corrected odds ratio signal score; F@1, F-score using a cutoff of 1 article; F@3, F-score using a cutoff of 3 articles; M+, number of positive test cases containing information in MEDLINE; M−, number of negative test cases containing information in MEDLINE.						
